# Quenchbodies That Enable One-Pot Detection of Antigens: A Structural Perspective

**DOI:** 10.3390/bioengineering10111262

**Published:** 2023-10-30

**Authors:** Hee-Jin Jeong

**Affiliations:** Department of Biological and Chemical Engineering, Hongik University, Sejong-si 30016, Republic of Korea; heejinjeong@hongik.ac.kr

**Keywords:** Quenchbody, immunoassay, Fluoroantibody, on-site assay, reagentless sensor

## Abstract

Quenchbody (Q-body) is a unique, reagentless, fluorescent antibody whose fluorescent intensity increases in an antigen-concentration-dependent manner. Q-body-based homogeneous immunoassay is superior to conventional immunoassays as it does not require multiple immobilization, reaction, and washing steps. In fact, simply mixing the Q-body and the sample containing the antigen enables the detection of the target antigen. To date, various Q-bodies have been developed to detect biomarkers of interest, including haptens, peptides, proteins, and cells. This review sought to describe the principle of Q-body-based immunoassay and the use of Q-body for various immunoassays. In particular, the Q-bodies were classified from a structural perspective to provide useful information for designing Q-bodies with an appropriate objective.

## 1. Quenchbody

Fluorescent immunosensors, which are produced by conjugating fluorescent probes to the antibody, have been widely studied owing to their ease of handling, high sensitivity, and rapid assay time [1,2]. Reagentless fluorescence immunoassay, which comprises homogeneous fluorescent antibodies, whose fluorescence changes due to binding to a target antigen, has been developed [3,4,5]. Reagentless fluorescence immunoassay requires only a few minutes to quantify the target antigen as the steps of washing, blocking, and incubation with secondary and/or tertiary antibodies are eliminated. Förster resonance energy transfer (FRET) is one of the conventional reagentless fluorescence immunoassays [6,7,8]. When donor and acceptor probes are nearby, the energy created by the emission wavelength of the donor is transferred to an adjacent acceptor. The fluorescence of an excited acceptor is stimulated, leading to an increase in its signal. This assay has limitations as the distance between the donor and acceptor must be precisely controlled to obtain a notable FRET effect [9]. FRET also requires a pair of fluorophores; however, the range of the fluorophore with non-overlapped emission spectra is limited [10]. As the change in a FRET signal before and after the addition of an antigen is low and sometimes even lower than the background signal, a ratiometrically calculated control experiment is required for FRET-based measurements. Another reagentless fluorescent immunoassay involves the fusion of fluorescent proteins and antibody-based approaches [11,12,13]. However, as the molecular weight (MW) of a fluorescent protein, such as green fluorescent protein (GFP), is large (28 kDa), the structure, production yield, and function of the original antibody can be affected. The antibodies are labeled using a small fluorescence dye (approximately MW 1000). For example, N-hydroxysuccinimide (NHS) ester-conjugated dye was conjugated to lysine (Lys) residues and the N-terminus primary amine group of the antibody [14]. However, because the labeling efficiency of this method depends on the number of Lys residues on the surface of the antibody, exact antigen quantification is difficult when Lys residues are blocked [15].

**Figure 1:**
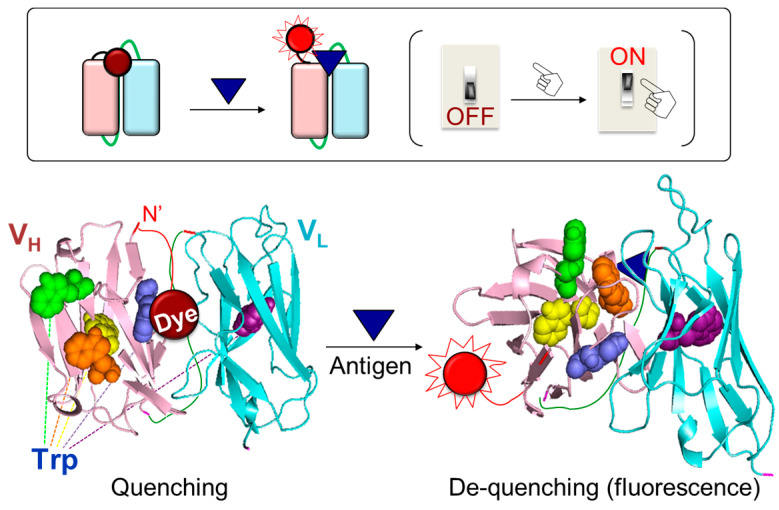
Reaction mechanism of the Q-body: antigen-dependent release of the quenching effect on a fluorophore.

A powerful reagentless fluorescent immunoassay reagent, Quenchbody (Q-body), was developed as an innovative technology to overcome the limitations of the above assays [16]. A Q-body is an antibody or its fragment that has a fluorescent dye incorporated at a specific site. The fluorescence intensity of Q-bodies increases upon antigen-dependent removal of the fluorophore via de-quenching. When a fluorescent dye is introduced around the paratope and is near the tryptophan (Trp) residues of the antibody, the fluorescence of the dye is quenched by Trps. Quenching is resolved after antigen addition as the antibody moves spatially to interact with the antigen, resulting in increased fluorescence emission (Figure 1) [17,18]. Ueda et al. discovered this phenomenon when they sought to improve the sensitivity of open sandwich FRET immunoassay for the detection of bone Gla protein (BGP), a biomarker of osteoporosis [19]. The researchers generated a variable heavy-chain (VH) domain and variable light-chain (VL) domain of anti-BGP antibodies, and labeled the N-terminus of each domain using red-colored tetramethylrhodamine (TMR) and green-colored rhodamine110 (R110) dyes. These dye-conjugated domains were mixed at the same molar ratio and the fluorescence intensity was measured following antigen addition. As fluorescence increased according to the antigen concentration, their original objective to develop an open sandwich FRET-based homogenous immunoassay was achieved. However, as the increased fluorescence value of the TMR was higher than the decreased fluorescence value of R110, the researcher opted to investigate this phenomenon. Briefly, the TMR-labeled VH and non-labeled VL domains were mixed, and then the antigen was added to the mixture. Surprisingly, the fluorescence intensity increased 1.7-fold, despite no FRET pairing [17]. Unlike FRET-based immunoassay, which requires precise adjustment of the distance between fluorophore pairs, the “single dye-labeled antibody” allows a fluorescence response with just one type of dye. The researchers named the novel concept of a “single dye-labeled antibody” a Q-body, conducted more detailed studies to identify the working mechanism, and expanded the application range of the Q-body. 

## 2. Reaction Mechanism of the Q-Body

Interestingly, the fluorescence of the Q-body increases in the presence of antigens, despite the presence of a single dye in the system. Prior studies provide hints regarding the observed fluorescence enhancement, as they describe the fluorescence quenching of boron-dipyrromethene (BODIPY) dye when introduced near a Trp or tyrosine (Tyr) residue [20,21]. Doose et al. found that quenching of the fluorophore is attributed to photoinduced electron transfer (PeT) from Trp to the dye [22]. As the indole side chain of Trp is the most readily oxidized functional group, Trp serves as an electron donor for PeT and the aromatic dye serves as an acceptor [23]. According to Ueda et al., a PeT-based immunoassay can be developed using conformationally flexible fluorophores conjugated to the antibody [16]. These researchers found that the observed fluorescence response of the “single dye-labeled antibody” was due to intramolecular quenching by Trps in the antibody. Thus, an antibody against BGP, which has four Trps in the VH domain and one in the VL domain, was employed as a model antibody. Thereafter, a three-dimensional structure of the anti-BGP Fab was created (PDB 5X5X), which revealed that three of the five Trps were located in a framework region, and two were located close to the VH-VL interacting surface. Two Trps were found to contribute to the hydrophobic interaction with the VL domain, and one contributed to the interaction with the BGP peptide antigen. Ueda et al. mutated each Trp to a phenylalanine, a non-quenching amino acid with a similar structure to Trp, and tested the effect of Trp on quenching [16,24]. The fluorescence response of the mutants was found to decrease compared to that of the wild type, suggesting that the fluorescent dye was located on the interface between VH and VL, and interacted with Trps, resulting in quenching via hydrophobic and/or π-π stacking interactions [25,26]. In the presence of antigens, the antigen bound to the antibody and induced movement of the VH and VL domains to stabilize the antibody conformation [27]. Therefore, the interaction between dye and Trps was weakened, resulting in de-quenching via the release of the dye. The conjugation of a dye near the paratope is crucial for obtaining high antigen-dependent responses because the dye is de-quenched when the antigen binds to the antibody. In cases where the dye is randomly bound to the antibody or conjugated to a position in far proximity from the antigen-binding site of the antibody, the interaction between the antigen and antibody hardly affects the movement of the dye [28]. Therefore, the release of quenching hardly occurs when the dye and antigen are far from each other, even if the dye is quenched, as it is located near Trps. Therefore, site-specific conjugation of the dye near the N-terminal region of the antibody is preferred because the antigen-binding site is close to the variable region of the antibody. In contrast, the conjugation of the dye in far proximity from the antigen-binding site of the antibody, such as the Fc region, is not suitable for generating high-performance Q-bodies. The antigen-binding activity of several Q-bodies was examined by enzyme-linked immunosorbent assay (ELISA). No significant differences were observed in the signals of the Q-bodies and the unlabeled antibody fragments, which suggests that fluorescence labeling had a negligible effect on the antigen-binding activity of the antibody [29,30,31]. An ELISA using anti-TMR antibodies and a Brownian motion-based fluorescence polarization assay supported the hypothesis that fluorescence enhancement is caused by the dynamic motion of the released and outbound dyes [32]. A molecular dynamics simulation was performed to further assess the antigen-dependent quenching mechanism of the Q-body via Trps [33]. The number and position of Trps are important for obtaining a high fluorescence response of Q-bodies. Based on the antibody sequence database (AbYsis) [34], the conservation of Trp in the antibody variable region is high (greater than 94%), indicating that most antibody variable regions have Trps. However, currently, not all antibodies can be converted to Q-bodies with a high response, which might be due to the structural accessibility of the dye to Trps. When a denaturant (guanidine hydrochloride and dithiothreitol) is added to a Q-body, the fluorescence increases to a level that is similar or higher than the saturated intensity in the presence of an antigen. This result indicates that the three-dimensional structure of antibodies is destroyed by the addition of a denaturant, and the distance between the dye and Trps increases, thereby eliminating quenching. 

Rhodamine dye is well known to be associated with quenching via Trps and/or Tyrs [16,20,35]; therefore, TMR, a type of rhodamine dye, is used at the initial stage of Q-body generation. Previously, three commercially available maleimide-conjugated rhodamine dyes, TMR, ATTO520, and R6G, with non-overlapping wavelengths, were used to generate a maleimide–thiol reaction for Q-body labeling [31,36,37,38,39,40,41,42,43,44]. Thereafter, more maleimide-conjugated commercial rhodamine dyes, including ATTO495 and Rhodamine Red, and non-rhodamine dyes, such as Cy3, ATTO590, ATTO655, and BODIPY series, were examined to obtain a high Q-body response [29,36,37,45,46]. Although the dye-dependent Q-body response could not be clearly identified, the different characteristics of dyes, such as shape, hydrophobicity, and lipophilicity, might be important [17,47,48]. 

## 3. Structural Types of Q-Body

### 3.1. Structures of IgG and Its Fragments

**Figure 2:**
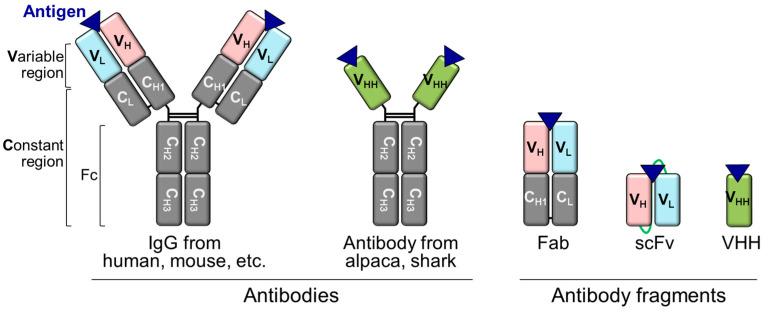
Structures of antibodies and antibody fragments.

**Figure 3:**
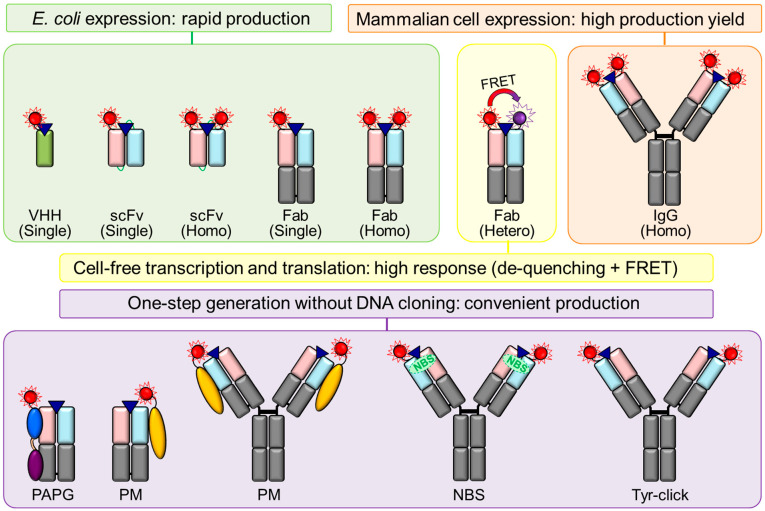
Various approaches for generating high-performance Q-bodies.

Immunoglobulin G (IgG) is a Y-shaped antibody generated by most animals, including humans, mice, rabbits, and goats (Figure 2). IgG consists of an antigen-binding region-containing VH and VL domains, constant regions of heavy chains (CH1, CH2, and CH3), and a constant region of light chain (CL). As complementarity-determining regions (CDRs) of antibodies, where antigens bind parts of the VH and VL domains, a combination of the VH and VL domains with other domains can be achieved via genetic recombination of the corresponding antibody fragment-encoding DNA [49]. scFv is formed from the VH and VL domains connected via a flexible peptide linker. As scFv is smaller (approximately 25 kDa) than IgG (approximately 150 kDa) and a fragment antigen-binding (Fab) antibody (approximately 50 kDa), it allows easier access to the highly concentrated target antigen, such as dense cancer cells, which is advantageous in cell penetration reactions [50,51]. Single domains, such as VH and VL, are the smallest IgG fragments but have half the number of CDRs relative to scFv. Therefore, their antibody-binding efficiencies are lower than that of scFvs. To date, various formats of Q-bodies against the antigens of interest have been developed (Table 1, Figure 3).

### 3.2. scFv-Type Q-Bodies

In the initial stage of Q-body development, scFv-type Q-bodies were generated using a cell-free protein transcription and translation system combined with a non-natural amino acid [16,24,36,37,45,54,60,65]. This method was based on the incorporation of fluorescent dye-conjugated aminoacyl tRNA into the amber, four-base (CGGG), or UAA codon at the scFv terminus [74,75,76]. For example, to generate a Q-body via cell-free transcription and translation with an amber suppression method, tRNA-conjugated rhodamine dye was incorporated into a ProX-tag, located at the N-terminus scFv (MSKQIEVNXSNE), which is an amber codon (X) containing an optimized sequence to enhance the incorporation of non-natural amino acids and increase protein expression yield [16,77]. A Q-body was generated by mixing the scFv with ProX-tag-encoding DNA, tRNA-fluorescent dye, and a transcription and translation reagent, which comprised amino acids and lysates. However, the high costs of tRNA-fluorescent dye and the cell-free transcription and translation reagent, and the limited production yield of this system prevent its widespread use *in cell* or *in vivo* experiments and its practical applications, in which a large quantity of probes is required. To overcome this issue, an *Escherichia coli*-based scFv-type Q-body was produced by post-labeling a recombinant scFv expressed from *E. coli* [29,40,56,57]. The ProX-tag for non-natural amino acid incorporation was substituted with a cysteine (Cys)-containing tag (Cys-tag, MSKQIEVNCSNE) or Srt-tag (GGGGG). The dyes for post-labeling, such as maleimide-conjugated dyes for maleimide–thiol reaction-based labeling [78] and LPETGG-conjugated dyes for Sortase-mediated labeling [79], are less expensive than tRNA-conjugated dyes and have a wider color range, thereby providing greater flexibility in the multiplex detection. Notably, the length of the spacer between the labeling tag and scFv was adjusted to increase the Q-body response [16,32,80].

### 3.3. Fab-Type Q-Bodies

Fab has two N-termini, one on each of the H and L chains, enabling separate conjugation of two dyes per Fab via the insertion of an amber and/or four-base codon on both N-termini. The response of double-TMR-labeled anti-BGP Fab-type Q-body with amber codons at the N-terminal of both H and L chains was higher than those of single-TMR-labeled Q-bodies with an amber codon at the N-terminal of either the H or L chain [37]. By using two different codons (that is, amber and four-base codons), two distinct dyes were conjugated to each H or L chain [81]. When the two N-termini of Fab were labeled using distinct dyes, a higher response was observed relative to that obtained via labeling with the same dye due to the addition of the FRET phenomena between the de-quenched dyes. For example, the fluorescence intensity of the doubly TMR-labeled Fab-type anti-BGP Q-body increased 25-fold; however, the response of the Fab-type anti-BGP Q-body, whose N-terminal on the H chain was labeled with TMR whereas that on the L chain was labeled with ATTO655, was improved 50-fold [37]. *E. coli*-based large-scale production of recombinant Fab, followed by its site-specific labeling via a maleimide–thiol reaction was performed to generate Fab-type Q-bodies against several antigens, including amyloid β oligomers, ICP, HER2, methamphetamine derivative, His-tag peptide, anti-His-tag antibody, TNFa, aldosterone, p53, SARS-CoV-2 spike protein, SARS-CoV-2 N protein, and 17β-estradiol (Table 2). The *E. coli*-based construction of double-labeled Fab-type Q-bodies was achieved by substituting the ProX-tag with a Cys-tag, and the number of labeling sites and/or the fluorescence dye were optimized to obtain a high response. Similar to scFv-type Q-bodies, commercially available maleimide-conjugated dyes with different excitation and emission wavelengths were conjugated to the Cys-tag of the Fab-type Q-body via a maleimide–thiol reaction. Dong et al. developed a transamination-based Fab-type Q-body by converting the N-terminus amine group of Fab to a ketone group to conjugate a fluorescent dye containing aminooxy or hydrazide groups on the ketone group [37,58]. To enable tandem affinity purification of the Fab-type Q-body, FLAG and His-tags were fused to the C-terminus of the H and L chains, respectively. When the purification efficiency was not high, not only the heterodimeric Fab with the same ratio of H and L chains but also free H/L chains were observed, resulting in a low sensitivity of the Q-body assays. The *E. coli*-based scFv-type Q-body against BGP led to higher production yields due to the lower expression of a Fab, whose structure was more complex than that of scFv, and the loss of target proteins during tandem purification. However, the thermal stability of the Fab-type Q-body was found to be higher than that of the scFv-type Q-body [29]. For some Fab-type Q-bodies double-labeled using the same fluorescent dye, homo-dimerization (H-dimer formation) was recognized to be affected, resulting in reduced sensitivity due to quenching between the two de-quenched dyes [37,82,83].

### 3.4. IgG-Type Q-Bodies

Cell-free- and *E. coli*-based Q-body generation required DNA construction followed by recombinant antibody expression before the labeling procedure. In particular, DNA cloning for insertion of the specific codon was necessary for inserting a labeling tag into the antibody-encoding sequence. However, if the sequence information for the antibody of interest has not been explored, animal immunization followed by hybridoma-based antibody production [84] or phage display-based antibody generation [85,86], and finally antibody sequence identification must be performed. As a result, a more simplified Q-body production method that directly converts both recombinant antibodies and commercially available antibodies into a Q-body without DNA manipulation and genetic fusion was established.

Site-specific fluorescent dye-labeled antibody-binding proteins, such as *Staphylococcus* protein A (PA), *Streptococcus* protein G (PG), *Mycoplasma genitalium* protein M (PM), and mouse IgG1-selective affibody, were generated as universal independent quenching and de-quenching probes to simply convert commercially available or synthesized antibodies to Q-bodies. PA binds to VH and Fc regions of IgG, and PG binds to the Fc and Fab regions of IgG [87,88,89,90,91]. Jeong et al. generated a PA-PG fusion protein by conjugating PA and PG using an optimized linker between the two proteins to increase their binding affinity [68]. A PA-based affibody called Zmab, which binds to mouse IgG1, was developed as a Q-body converting probe [68]. Thereafter, Miyake et al. established a PM-based Q-body converting method. PM strongly binds to the L chain, predominantly at the VL end, of almost all antibodies, and its binding affinity is higher than those of PA and PG due to its wider antibody-binding area [92]. The researchers conjugated a fluorescent dye to the PM using the maleimide–thiol method and found that the “PM Q-probe” converted Fab- or IgG-type antibodies to Q-bodies within a few minutes [44,55]. Evidently, this fusion protein-based Q-body generation was valuable in reducing the time and effort needed to convert a native antibody or its fragment without a labeling tag to Q-bodies. An IgG-type Q-body was also generated via ultraviolet (UV)-based photochemical crosslinking of an indole-3-butyric acid (IBA)-conjugated fluorescent dye to the nucleotide-binding sites (NBSs) near the antigen-binding sites [93,94,95]. Jeong et al. established the NBS-based photochemical crosslinking strategy to conjugate a fluorescent dye to an antibody in a one-step procedure that did not require DNA cloning; a covalent bond was formed between NBS and IBA via UV irradiation, resulting in a Q-body [53]. Hohsaka et al. performed reductive alkylation under weak acidic conditions (pH 5) to produce an IgG-type Q-body [65]. N-terminal-selective fluorescent labeling was carried out in the presence of IgG, aldehyde-derivatized dye, and picoline borane. Thereafter, the researchers validated the aldehyde-derivatized dyes and the alkyl linker length. The pH value was found to influence fluorescence intensity, and labeling had no impact on IgG binding affinity. Sato et al. developed Q-bodies using pharmaceutical anti-HER2 IgG (trastuzumab) and an anti-cluster of differentiation 20 (CD20) IgG (rituximab) via tyrosine chemical modification [46]. Full-length IgGs were modified into Q-bodies using CDR-selective Cu-free click reactions that selectively label the exposed Tyrs of the protein. Recently, recombinant IgGs against the SARS-CoV-2 spike protein or FLAG peptide were manufactured using HEK293F cells and their maleimide–thiol reaction-based conversion to Q-bodies were revealed [42,66].

### 3.5. VHH-Type Q-Bodies

VHH is the smallest (approximately 15 kDa) antibody fragment of heavy-chain-only antibodies generated from alpacas and sharks but has the same antigen-binding regions as full-length heavy-chain-only antibodies (approximately 90 kDa) [96,97]. Inoue et al. generated VHH-type Q-bodies against methotrexate (MTX) via maleimide–thiol reaction-based labeling of VHH expressed from *E. coli* by optimizing the fluorescence dye and spacer length [69]. A superior VHH-type anti-MTX Q-body was then selected from the antibody library via a yeast surface display using fluorescence coiled-coil interaction [59] and flow cytometry [70]. The yeast surface display-based VHH-type Q-body screening was also employed to quantify human serum albumin (HSA) [70], and VHH-type Q-bodies against lysozyme with a large variation of fluorescence dye were generated [31]. 

### 3.6. Protein-Type Q-Bodies

The Q-body mechanism was applied to discover a rapamycin-mediated interaction between FKBP12 and FRB via site-specifical conjugation of fluorescence dyes to proteins [36]. A protein-type Q-body concept was applied not only to protein-protein interactions but also ligand-receptor interactions. Li et al. generated a protein-type Q-body by conjugating a fluorescent dye to the N-terminus Cys-tag of recombinant programmed cell death-1 (PD-1) proteins via a maleimide–thiol reaction [72]. These researchers used the fluorescent PD-1 protein to detect its ligand, programmed cell death-ligand 1 (PD-L1), using the interaction between PD-1 and PD-L1. 

### 3.7. Luciferase-Conjugated Q-Bodies

NanoLuc (NLuc), an engineered luciferase [98], was fused to a Q-body. The “BRET Q-body” exhibited improved response and sensitivity relative to a Q-body, owing to the bioluminescence resonance energy transfer (BRET) principle, which was combined with the quenching/de-quenching mechanism [73]. In the absence of antigens, the dye was quenched and the luminescence of NLuc increased after the addition of a luminescent substrate. In the presence of antigens, the dye is released; thus, BRET occurs, resulting in increased fluorescence intensity of the dye. As an external excitation light source was not required, visual observation of the color change could be performed [99]. Moreover, photobleaching due to a strong excitation light did not have to be considered [100,101].

### 3.8. Structural Strategies for Improving the Fluorescence Response of Q-Body

Optimization of the spacer length between fluorophore and antibodies, such as the flexible GS linker length, and the distance between fluorophores and maleimide was effective in improving the fluorescence response of various Q-bodies [16,32,40,69]. For example, the fluorescence response of TMR-C0-, TMR-C2-, TMR-C5-, and TMR-C6-maleimide-conjugated Q-bodies varied [36]. Moreover, during transamination-based Q-body generation, aminooxy-5(6)-TMR and TMR-C5-peptide-hydrazide, which is the longer of the two, resulted in different responses [58]. The location and number of labeling sites are crucial in the generation of a superior Q-body [36]. When tRNA-R6G dye was incorporated into an N-terminal region of anti-PS82 scFv (VH-VL), the intensity in the presence of antigens increased 1.2-fold [54]. However, when the orientation of scFv was changed by reversing the VH-VL to VL-VH, the signal improved 2.1-fold. Four Trps exist in the VL of anti-PS82 antibody and one Trp exists in the VH. Therefore, the dye attached to the N-terminus of VL-VH may have a higher chance of quenching than that attached to the N-terminus of VH-VL. When another amber codon was added not only to the N-terminal region but also in the middle of the inter-domain linker region and two R6Gs were conjugated, the doubly R6G-labeled VL-VH type Q-body exhibited a significant fluorescence of 6.7-fold. Adjustment of the number and position of fluorophores was also important for constructing Q-bodies against HEL, Nef, or FLV [17,40,45]. 

## 4. Q-Bodies against Various Antigens of Interest

### 4.1. Anti-BGP Q-Body as a Model Q-Body

Human bone g-carboxyglutamic acid-protein (BGP) is a major non-collagen bone protein and a biomarker for bone metabolism, insulin regulation, and male fertility [102,103]. The anti-BGP antibody was used to create the first Q-body as its conformation was markedly stabilized by BGP or BGP peptides, including C-terminal valine epitope (BGP-C7; RRFYGPV and BGP-C10; EAYRRFYGPV) [104]. Thereafter, the anti-BGP Q-body was used as a model Q-body for developing novel generation approaches and comparing properties. At first, TMR-conjugated scFv-type anti-BGP Q-body was generated using a cell-free system. The fluorescence spectrum revealed a BGP-C7 concentration-dependent response [16]. The EC50 values for the scFv-type anti-BGP Q-body and the non-labeled scFv obtained using competitive ELISA were 2.5 × 10^−8^ M and 8.8 × 10^−8^ M, respectively, indicating that the antigen-binding activity of scFv was maintained after dye incorporation at the N-terminal region, which is close to the antigen-binding site [104]. The response of the anti-BGP Q-body against not only BGP-C7 in PBS (5.6-fold) but also BGP-C10 (5.8-fold), human BGP (5.2-fold), and BGP-C7 in 50% plasma (7.0-fold) was revealed, resulting in similar responses [16]. The fluorescence responses of anti-BGP Q-bodies varied according to the antibody type, labeling method, and number and type of conjugated dyes (Table 2). 

### 4.2. Q-Bodies against Small Molecules

Q-body systems have been used to detect various small molecules, including BGP peptide, intact BGP, 17β-estradiol, illegal drugs such as codeine, heroin, morphine, DON, fluvoxamine, progesterone, testosterone, thyroxine 4, cortisol, His-tag peptide, aldosterone, digoxin, and FLAG peptide (Table 3). As antigens, such as hapten and peptide, have a low molecular weight and are monovalent with a single-epitope, sandwich ELISA is generally difficult to perform as two epitopes are required in an antigen for binding to two types of antibody [105,106]. Moreover, indirect and direct ELISAs have limited use for small molecule detection as the immobilization of antigens to the surface of a 96-well plate is challenging due to its small binding area. Therefore, competitive ELISA is generally used to detect low-molecular-weight antigens; however, careful manipulation of the concentrations of antibodies, antigens, and detecting probes is required to obtain a high S/B. A detecting probe, such as enzyme- or fluorescent dye-labeled antigens, should also be generated or purchased as a competitive molecule, which is labor-intensive and less cost-effective. In contrast, detecting probe is not required for antigen competition and a carrier protein is not required for antigen immobilization to perform the Q-body-based assay, which is remarkedly distinguishable from other conventional immunoassays for detecting small molecules.

### 4.3. Q-Bodies against Proteins

Large proteins, such as hen egg lysozyme (HEL) [16,17,31], BSA [16], HSA [16], Nef [45], TNFa [41,80], SARS-CoV-2 spike protein [42], and SARS-CoV-2 N protein [44], have been demonstrated to be target antigens for Q-bodies. Trps in the protein antigen affect the fluorescence response itself; therefore, the saturated intensity in the presence of a high concentration of antigens cannot reach that of fully denatured Q-bodies. Thus, re-quenching of the de-quenched fluorescence can be carried out by the Trps of the protein antigen. For example, the fluorescence response in the presence of antigens, and the three-dimensional homology modeling results of Q-bodies against HEL, which comprised three Trps, indicated the re-quenching of the de-quenched fluorophore by the Trp(s) of HEL [17]. Moreover, if not enough space is available for dye movement between the antigen and antibody due to the size of the antigen, de-quenching of the dye may barely occur. When the large antigen interacts with the antibody and is located to the antibody, the dye moving out of the antibody can be re-inserted into or hindered from reattaching to the antibody or can still be located near the Trps, resulting in re-quenching. 

### 4.4. Q-Bodies against Cells

Q-bodies have been used for cellular imaging. For example, a double-ATTO520-conjugated Fab-type Q-body was used for the microscopic observation of BGP on osteosarcoma cells induced for osteoblast differentiation [37]. Jeong et al. developed Q-bodies to image membrane-protein clauidin4 (CL4) on the surface of human myoblast HT-1080 cancer cells [38]. The cells displayed distinct cell-surface staining with low background fluorescence. When the anti-CL4 Q-body was compared with a AF488-conjugated anti-CL4 antibody, which was labeled via conventional NHS coupling, lower background fluorescence was observed from the Q-body without the time-consuming and labor-intensive washing processes. One-step imaging of HER2-positive cell strains, A549 lung carcinoma, and SK-BR3 breast adenocarcinoma were successfully performed using anti-HER2 Q-bodies [61]. The staining pattern was almost similar to that observed using conventional immunostaining, which required a longer assay time, including washing. Q-bodies based on trastuzumab and rituximab were generated and their fluorescence responses against CD20+ SU-DHL-4 and HER2+ SK-BR-3 cell lysates were confirmed [46]. Dai et al. developed a Fab-type Q-body that targets p53 peptide, which is a key tumor suppressor and validated biomarker for cancer diagnostics and therapeutics [57]. These researchers spatiotemporally visualized intracellular p53 in fixed and living cells (such as HCT116, SK-BR-3, and WiDr) and suggested that the Q-body could be used for intracellular antigen-specific live-cell sorting.

## 5. Conclusions

Q-bodies are powerful reagentless homogeneous biosensors with advantages, such as rapid detection and low handling requirements, as they do not require immobilization or washing. In this review, we classified Q-bodies based on their structures and summarized their broad application for detecting various target antigens from small haptens and peptides to large proteins and cells. Since the first Q-body concept was revealed in 2011, several ideas to increase fluorescence response and screen high-performance Q-bodies have been presented. The responses of certain Q-bodies do not reach industrial or in vivo detection levels. Molecular evolution is a promising method for obtaining Q-bodies with high quenching and de-quenching efficiencies. For example, a Trp-rich antibody library generated via display technology, such as yeast display and mRNA display, can be utilized to generate mutated antibodies, where large amounts of Trps are located near the antigen-binding region. Screening for the optimal engineered antibody with the same or elevated antigen-binding efficiency after CDR mutation and that exhibits increased quenching efficiency following the addition of Trps followed by the release of quenching in the presence of antigens can be an efficient approach toward generating a selected Q-body. Moreover, molecular dynamics simulations can be utilized to design a Q-body. Through in silico antibody-antigen docking simulation, a computationally predicted Q-body sequence that selectively recognizes and strongly binds to the epitope of the antibody can be designed. In addition to these pre-selections, post-selections, such as FACS sorting and droplet microfluidic single-cell analysis, are suitable for increasing the sensitivity and response of the Q-body. Because it has antigen-binding properties and is smaller than IgG, scFv has relatively high cell-penetration ability [107,108,109]. When a large fluorescent probe, such as a fluorescent protein, is conjugated to an antibody or antibody fragment, the size of the fusion antibody increases, causing steric hindrance or obstructing the recognition of epitopes on the cell surface [110,111]. Therefore, the scFv-type Q-body, where an organic dye with a MW of approximately 1000 is conjugated, is theoretically preferred for generating a Q-body, which is especially utilized for cellular diagnosis. In contrast, the half-life of scFv is shorter than that of IgG, because the Fc region plays a crucial role in extending the half-life of the antibody by interacting with neonatal Fc receptors (FcRn) in the human body [112,113]. Therefore, the scFv-type Q-body may be relatively suitable for in vitro and ex vivo diagnosis, such as lateral flow immunoassay, fluorescence spectrometry measurement, flow cytometry assay, and microscopy imaging, and the IgG-type Q-body is preferred for in vivo diagnosis, such as the in vivo imaging system (IVIS), which injects the Q-body into the animal model and monitors its live imaging. A greater color variation of usable fluorophores will improve the performance of Q-bodies towards the simultaneous detection of multiple antigens in situ. To further investigate its versatility for broad applications in biosensing and biological imaging, we expect more suitable strategies for most antibodies of interest to be investigated. 

## Figures and Tables

**Table 1:** Various types of Q-bodies.

Q-Body Type	Antigen	Reference
scFv	Bone gla protein (BGP) or BGP peptide	[16,29,32,52,53]
	Bisphenol A	[16,32]
	Hen egg lysozyme (HEL)	[16,24]
	Bovine serum albumin (BSA)	[16]
	Human serum albumin (HSA)	[16]
	Estradiol	[16]
	Morphine	[16]
	Heroin	[16]
	Codeine	[16]
	Phosphorylated vimentin peptide	[54]
	HIV antigen Nef	[45]
	Fluvoxamine	[40]
	Cortisol	[55]
	His-tag	[56]
	p53 tumor suppressor	[57]
Fab	BGP or BGP peptide	[37,58,59]
	HSA	[37]
	Morphine	[37]
	Methamphetamine	[32,37]
	Cocaine	[37]
	Deoxynivalenol	[32,60]
	Claudin4	[38]
	Amyloid β oligomer	[39]
	HER2+ cells	[61]
	His-tag	[56]
	Tumor Necrosis Factor-α	[41]
	Aldosterone	[62]
	p53 tumor suppressor	[57,63]
	SARS-CoV-2 S1 protein	[42]
	SARS-CoV-2 pseudovirus	[42]
	SARS-CoV-2 N protein	[44]
	Digoxin	[43]
	17β-estradiol	[64]
IgG	HSA	[53]
	FLAG-tag	[65,66]
	His-tag	[65]
	HA-tag	[65]
	Thyroxine	[65]
	Testosterone	[67]
	CD20+ cells	[46]
	HER2+ cells	[46]
	SARS-CoV-2 S1 protein	[42]
PAPG	BGP or BGP peptide	[68]
	Vimentin	[68]
PM	BGP or BGP peptide	[55]
	Progesterone	[55]
	Testosterone	[55]
	Thyroxine4	[55]
VHH	Methotrexate	[59,69,70]
	HSA	[70]
	HEL	[31]
	Quinalphos	[71]
Protein	Rapamycin	[36]
	PD-L1	[72]
Luciferase-fusion	BGP	[73]

**Table 2:** Fluorescence responses of the anti-BGP Q-bodies. n.d. means not described.

Antibody Format	Labeling Method	Dye (Position)	Response (Fold)	EC50 (nM)	Reference
scFv	Cell-free	TMR-C6	5.6	25	[16]
		TMR	5.8	38.0	[54]
		R6G	7.7	84.6	[54]
		AT520	2.9	17.7	[54]
	Maleimide–thiol	TMR-C0	2.9	n.d.	[29]
		TMR-C2	2.0	n.d.	[29]
		TMR-C5	4.0	4.4	[29]
		AT495	1.1	n.d.	[29]
		AT520	2.7	6.3	[29]
		R6G	5.0	11.4	[29]
		Rho	1.7	70.9	[29]
	NBS	IBA-C8-TMR	9	116.8 ± 32.0	[53]
	BRET	AT520	1.9	n.d.	[73]
		R6G	4.8	n.d.	[73]
		TMR	7.2	n.d.	[73]
Fab	Cell-free	TMR (H)	9.6	21	[37]
		TMR (L)	1.8	7.1	[37]
		TMR (H), TMR (L)	21.2	10,000	[37]
		R110 (H)	1.1	7.8	[37]
		R110 (L)	1.3	7.2	[37]
		R110 (H), R110 (L)	2.4	7.6	[37]
		AT655 (H)	4.6	4.6	[37]
		AT655 (L)	2.7	9.8	[37]
		AT655 (H), AT655 (L)	10.6	44	[37]
		TMR (H), R110 (L)	2.7	8.8	[37]
		R110 (H), TMR (L)	19	110	[37]
		TMR (H), AT655 (L)	35	61	[37]
		AT655 (H), TMR (L)	50	110	[37]
	Maleimide–thiol	AT520 (H, L)	11	77	[37]
		AT520	3.6	n.d.	[37]
		TMR-C5 (H, L)	2.4	n.d.	[37]
		TMR-C5	7.0	19	[37]
	Transamination	TMR-C5	2.75	n.d.	[58]
		Aminooxy-5(6)-TMR	1.70	n.d.	[58]
	PM	TMR-C6	2.0	81 ± 8	[55]
	Coiled-coil	TMR	2.7	17	[46]
IgG	PM	TMR-C6	~1.9	7.3 ± 0.6	[55]

**Table 3:** Q-bodies against various antigens of interest. When the same target antigen but different production methods was employed, maximum response was obtained among the Q-bodies. n.d. means not described.

Target Antigen	Antibody Format	Maximum Response (Fold)	EC50	LOD	Reference
Bisphenol A	scFv	2.0	20 nM	n.d.	[16]
BSA	scFv	1.5	n.d.	n.d.	[16]
17β-estradiol	scFv	4.5	n.d.	n.d.	[16]
Codeine	scFv	~1.5	19 nM	n.d.	[16]
Heroin	scFv	~1.5	37 nM	n.d.	[16]
Vimentin PS71	scFv	3.95 ± 0.4	n.d.	n.d.	[54]
Vimentin PS82	scFv	6.7 ± 0.2	3.8 nM	n.d.	[54]
HSA	Fab	7.8	n.d.	n.d.	[37]
Influenza HA protein	Fab	7	n.d.	n.d.	[37]
Morphine	Fab	7.2	n.d.	n.d.	[37]
Methamphetamine	Fab	7.2	n.d.	n.d.	[37]
Cocaine	Fab	3.5	n.d.	n.d.	[37]
HEL	VHH	~4.5	397 nM	n.d.	[31]
DON	Fab	3.9	101 nM	5000 nM	[60]
Rapamycin	Protein	1.5	3.6 nM	0.65 nM	[36]
Claudin	Fab	3.0	n.d.	1.9 ± 0.7 nM	[38]
Amyloid β peptide	Fab	2.2	1090 ± 40 nM	200 nM	[39]
ADDL	Fab	2.8	33,000 ± 26,000 nM	300 nM	[39]
Bio-DAE10	Fab	2.1	n.d.	n.d.	[39]
FLV	scFv	1.5	31.4 nM	n.d.	[40]
HER2	Fab	3.6	1.1 nM	0.08 nM	[61]
Progesterone	IgG	~1.3	110 ± 4 nM	0.96 nM	[55]
Testosterone	IgG	~2.0	1.37± 0.05 nM	0.35 nM	[55]
Thyroxine T4	IgG	~2.8	15.8 ± 1.2 nM	4.1 nM	[55]
Cortisol	scFv	2.46	45,200 nM	n.d.	[55]
Methotrexate	VHH	6.33	37.6 ± 11 nM	0.56 nM	[69]
c-Myc	scFv	2.2	n.d.	n.d.	[33]
Thyroxine T3	IgG	1.3 ± 0.05	n.d.	n.d.	[65]
FLAG	IgG	9.5 ± 0.5	n.d.	n.d.	[65]
hPDL1	Protein	1.43	110 nM	0.1 nM	[72]
Nef (HIV protein)	scFv	2	n.d.	n.d.	[45]
His-tag peptide	Fab	~5	132,000 ± 34,000 nM	3500 nM	[56]
CD20	IgG	~3.5	n.d.	n.d.	[46]
Tumor necrosis factor α	Fab	4.05	65.6 ng/mL	0.419 ng/mL	[41]
Aldosterone	Fab	5	42.3 ng/mL	24.1 pg/mL	[62]
p53	Fab	27	60 nM	0.72 nM	[57]
SARS-CoV-2 spike protein	Fab	~1.5	n.d.	0.11 nM	[42]
SARS-CoV-2 nucleocapsid protein	Fab	n.d.	n.d.	0.191 nM	[44]
Digoxin	Fab	2.2	0.256 ng/mL	0.023 ng/mL	[43]

## Data Availability

Data available on request from the author.
